# Políticas públicas de promoción de salud en el contexto de la COVID-19, en Chile, una aproximación desde el análisis situacional

**DOI:** 10.1177/1757975920978311

**Published:** 2020-12-18

**Authors:** Mirliana Ramírez-Pereira, Ricardo Pérez Abarca, Felipe Machuca-Contreras

**Affiliations:** 1 Departamento de Enfermería, Universidad de Chile, Santiago, Chile; 2 Universidad Autónoma de Chile, Santiago, Metropolitana, Chile

**Keywords:** infecciones por coronavirus, investigación cualitativa, política pública, teoría fundamentada, participación de la comunidad

## Abstract

**Introducción:**

el 18 de octubre del 2019, Chile vivió el estallido social más grande desde el retorno a la democracia en 1988. En marzo se esperaba retomar con mayor fuerza las movilizaciones, pero con la confirmación del primer caso de COVID-19, el Gobierno de Chile inició una serie de medidas de tipo preventivo, diferentes a las definidas por otros países.

**Metodología:**

se presenta un análisis de las políticas públicas de promoción de salud adoptadas por Chile en el contexto de la COVID-19. Para esto se utiliza el análisis situacional de Adele Clarke, que incorpora la construcción de mapas situacionales, de mundos sociales y mapas de posición. Para efectos de esta investigación se construye el mapas situacional. Se incluye en el análisis documentos oficiales del Ministerio de Salud, discurso del Presidente de Chile, discursos del Ministerio de Salud.

**Resultados:**

las medidas adoptadas por el Gobierno de Chile incorporan alcances del tipo de cuarentenas parciales y controles sanitarios, estado de excepción constitucional, toque de queda, plan económico de emergencia, salud, educación, comercio, transporte, control de fronteras, eventos masivos, vacunación contra la influenza y adultos mayores.

**Conclusiones:**

según el análisis realizado, se observa un eje transversal de prioridades de medidas de tipo económico, con todas las implicancias sanitarias, sociales y políticas que se derivan.

## Introducción

Los primeros casos de esta nueva enfermedad fueron reportados en Wuhan (China) en diciembre del 2019 presentándose como neumonías atípicas. Más tarde se identificó un nuevo coronavirus, el SARS-CoV-2, el cual se relacionó con la nueva patología denominada COVID-19 (1).

En enero del 2020, la Organización Mundial de la Salud (OMS) emitió una alerta global declarando la COVID-19 como una emergencia de salud pública de importancia internacional (2). Al 1 de julio del 2020, la OMS informaba 5218.590 casos confirmados y 249.318 fallecidos en la región de las Américas (3).

En Chile el 2 de marzo de 2020 se notificó el primer caso de persona portadora de SARS-CoV-2. Al 1 de julio el Ministerio de Salud de Chile (MINSAL) reportaba un total de 282.043 casos acumulados y 5.753 personas fallecidas (4).

La pandemia de la COVID-19 se ha convertido en una crisis sanitaria, con repercusiones sociales, económicas, políticas, éticas, de género y cuidados, las que impactan de sobremanera a los grupos más vulnerables.

Las decisiones que se toman en situaciones de crisis se encuentran vinculadas a las atribuciones de los Estados, las cuales son reguladas por la Constitución Política, que dicta las normativas que determinan las estrategias adecuadas. En el caso de Chile, la Constitución Política de 1980, instaurada bajo la dictadura cívico-militar de Augusto Pinochet y que aún sigue vigente, establece el rol Subsidiario del Estado, que se concretiza en dos principios: la focalización del gasto social dirigido a sectores desfavorecidos y la privatización de la educación, salud, previsión y vivienda (5), esto queda evidenciado con la inexistencia de mecanismos de protección y garantía directa del derecho a la salud y con protección reforzada para la libertad de empresa (6).

Como consecuencia de la privatización de la salud en Chile, el sistema quedó conformado por el sector público, representado por el Fondo Nacional de Salud (FONASA), que da atención al 70% de la población y a las fuerzas armadas, con un 10%. El sector privado, incorpora a la población con mayores recursos económicos equivalente a un 17,5%, a través de las Instituciones de Salud Previsional (7).

La libertad económica ha tenido un rol protagónico en las decisiones adoptadas para el enfrentamiento de la pandemia. Chile es uno de los países con mayor libertad económica del mundo, ubicado en el lugar quince a nivel mundial según el Índice de Libertad Económica 2020 (8).

La unidad de análisis de esta investigación se basó en las políticas públicas, las cuales se comprenden como los proyectos y actividades que un Estado diseña y gestiona a través de un gobierno y una administración pública a fin de satisfacer las necesidades de una sociedad (9). La autoridad de un Estado debe estar investida de poder público y legitimidad. La legitimidad está dada por el marco legal, según el cual, el Estado ejerce sus facultades a través de leyes y por medio del interés del bien público, que constituye una actividad esencial, mínima e indelegable (10).

Para lograr legitimidad, es necesario el actuar ético de los gobiernos, ya que permite un mejor desempeño en el marco del contenido normativo y prescriptivo. Cuando hay un sentido ético, la administración se compromete a una mejor forma de gobierno, funcional de la vida de los ciudadanos y las organizaciones sociales (11).

La cuarentena, el cierre de escuelas y el teletrabajo, ha repercutido en la vida de las mujeres, asumiendo el cuidado de hijos, personas mayores y en situación de discapacidad y las tareas del hogar, todo esto aparejado con el teletrabajo. Joan Tronto define el cuidado como una actividad de la especie, que incluye todo lo que se hace para mantener, continuar y reparar el mundo de tal forma que sea posible vivir en él. En sociedades más democráticas e igualitarias el cuidado representa el eje de muchas políticas públicas que permiten una mayor participación de la mujer y de todos los actores que no son habitualmente escuchados (12,13).

Como consecuencia de las atribuciones del Estado de Chile, con un rol principalmente de subsidiariedad y enmarcado en el libre mercado, han existido limitantes en la toma de decisiones respecto a la pandemia. A esto se suma, el cuestionamiento a la legitimidad del actual gobierno por su actuar durante el estallido social de octubre del 2019, en el cual la ciudadanía se alzó en protesta contra la desigualdad, y la falta de acceso a salud, a educación y pensiones dignas (14). La policía reprimió a la ciudadanía, cometiendo violaciones a los derechos humanos, según consta en los informes de Amnistía Internacional, la Oficina del Alto Comisionado de las Naciones Unidas para los Derechos Humanos y Human Rights Watch (15–17). Con la credibilidad cuestionada, el gobierno se ha enfrentado a una desconfianza generalizada a las instituciones y a una ciudadanía con un alto grado de vulnerabilidad (18).

En el contexto antes descrito, surge como un imperativo el estudio de las políticas públicas en torno a la pandemia con el propósito de producir nuevo conocimiento, que permita sobrellevar de mejor manera las futuras catástrofes y calamidades, tanto de tipo natural, como las provocadas por el ser humano.

Esta investigación tuvo como objetivo comprender los mundos sociales y escenarios de acción en las políticas públicas de promoción de la salud en Chile en tiempos de la COVID-19. El problema de investigación, redactado en forma de pregunta fue: ¿Cuáles son los mundos sociales y escenarios de acción en las políticas públicas de promoción de salud en Chile en los tiempos de la pandemia COVID-19?

## Material y método

La metodología en esta investigación fue de tipo cualitativa. Esta metodología permite interpretar las diferentes miradas, lecturas y símbolos de la realidad, aproximándose de esta forma a los diferentes contextos y marcos conceptuales, haciendo posible la organización, sistematización e interpretación de las políticas públicas (19,20).

Esta investigación se realizó bajo el paradigma postpositivista, en el cual el/la investigador/a se posiciona como observador/a relativo/a y neutral frente al fenómeno de investigación, aceptando la imposibilidad de alcanzar un estado objetivo absoluto al plantearse la realidad como externa (21).

La recolección de datos consideró el periodo comprendido entre el 30 de enero del 2020, fecha en que la OMS declaró la COVID-19 como Emergencia de Salud Pública de Importancia Internacional (ESPII) hasta el 30 de mayo del 2020. Se efectuó en dos etapas; en la primera de ellas se realizó una consulta virtual por medio de un formulario de Google tipo encuesta a 10 expertos y expertas del mundo académico y del sector público, los criterios de inclusión fueron definidos por los investigadores con base en voces expertas en epidemiología, políticas públicas, salud pública y ciencias sociales. Se les consultó acerca de las políticas que a su juicio eran relevantes en la pandemia y qué actores individuales y colectivos se encontraban presentes y ausentes en su desarrollo. En la segunda etapa se obtuvo información mediante un muestreo propositivo cuyos criterios de inclusión fueron documentos, programas, informes, sitios web y noticias relacionadas con COVID-19 en Chile provenientes de fuentes digitales oficiales, como son el consejo asesor, mesa social, departamento de epidemiología del Ministerio de Salud, desde medios, discursos y noticias ciudadanas y por medio del software Chorus Analytics _T.M_ con el cual se recogieron los actores relevantes y sus discursos, determinados por frecuencias de menciones en Twitter.

Se utilizó un enfoque deductivo para extraer y analizar datos, conformando categorías, las cuales fueron trianguladas por los autores, quienes poseen formación de posgrado en ciencias y políticas públicas. Como criterio de rigor se utilizaron los definidos por Treharne y Riggs: triangulación de datos entre investigadores, con el fin de explorar convergencias, complementariedad y disonancias, transparencia en el proceso investigativo y reflexividad durante el proceso (22).

El análisis se realizó según el modelo de análisis situacional, desarrollado por Adele Clarke, el cual utiliza la teoría fundamentada con el fin de identificar y descubrir los mundos sociales y escenarios de acción utilizando la representación por medio de mapas, de esta forma se logra una mirada integral de las conexiones y relaciones que influyen en los problemas sociales. El Mapa situacional permite comprender diferentes niveles de ordenamiento, dirigido a dibujar la situación con sus elementos históricos, culturales, simbólicos, materiales, humanos y no-humanos. El Mapa de mundos y arenas sociales identifica los diferentes grupos involucrados en la situación analizada y las relaciones entre los mismos. Por su parte, el Mapa de posición tiene como objetivo dar cuenta de las distintas posiciones discursivas presentes en la situación analizada (23).

La pandemia está presente en todo el planeta y no existen certezas respecto a su término. En el caso de Chile, los servicios de salud se encuentran saturados, las Unidades de Cuidados Críticos cuentan con un número limitado de camas, personal de salud y ventiladores mecánicos. Hasta la fecha de escritura de este artículo, no está incorporado el nivel de atención primario como un actor fundamental en la prevención, pesquisa precoz y tratamiento de la COVID-19, dado que las políticas públicas, inyección de recursos, como por ejemplo en la compra de respiradores artificiales y protocolización se han centrado en el fortalecimiento de la red asistencial hospitalaria de atención en salud (24). Considerando estos antecedentes, para efecto de esta publicación se presenta el Mapa situacional, siguiendo la recomendación de Clarke de utilizar como nombres de cada categoría los siguientes encabezados: actores individuales, actores colectivos, construcción de discursos de los actores individuales/colectivos, elementos o actores no humanos, elementos o actores implicados en silencio, construcción de discursos de actores no humanos, elementos políticos/económicos, elementos temporales, debates o temas relevantes, elementos socioculturales/simbólicos, elementos espaciales, discursos relacionados de tipo históricos (25).

Los aspectos éticos involucrados fueron *valor social o científico*, esperamos que esta investigación pueda ser un aporte en la sistematización de las políticas públicas por el Gobierno de Chile en el contexto de la pandemia COVID-19, así como también contribuya al levantamiento de información que pueda ser utilizada para futuras decisiones y reflexiones enfocadas en preservar la vida y la calidad de vida de las personas. También se consideró el criterio de *validez científica* con un riguroso diseño metodológico siguiendo las directrices del Análisis situacional, pocas veces utilizado en investigación en español, lo que esperamos pueda ser orientador para futuros estudios. También se utilizó literatura actualizada y el análisis fue triangulado por los investigadores. En relación con la p*roporción favorable de riesgo-beneficio*, esta investigación no tiene seres humanos como sujetos de estudio, sino documentos, desde ese punto de vista el riesgo es mínimo. Al mismo tiempo, el beneficio es mayor que el riesgo considerando el aporte social y la validez científica antes mencionada (26).

## Resultados

En la primera fase de recolección de información emergieron como relevantes, las políticas públicas de cuarentenas parciales y controles sanitarios, estado de excepción constitucional, toque de queda, plan económico de emergencia, salud, educación, comercio, transporte, control de fronteras, eventos masivos, vacunación contra la influenza y adultos mayores, además de los actores sociales individuales y colectivos presentes en la pandemia. De estos se seleccionaron, según criterio de relación directa en promoción y prevención de la COVID-19, las siguientes políticas: Cuarentenas parciales y controles sanitarios, salud, eventos masivos, educación, plan económico de emergencia y vacunación y adultos mayores.

En la segunda fase de recolección de datos, emergieron los actores individuales, colectivos, discursos de los diferentes actores, elementos o actores no humanos, en silencio, elementos políticos/económicos, elementos socioculturales/simbólicos, elementos espaciales, discursos históricos, entre otros.

**Figura 1.**
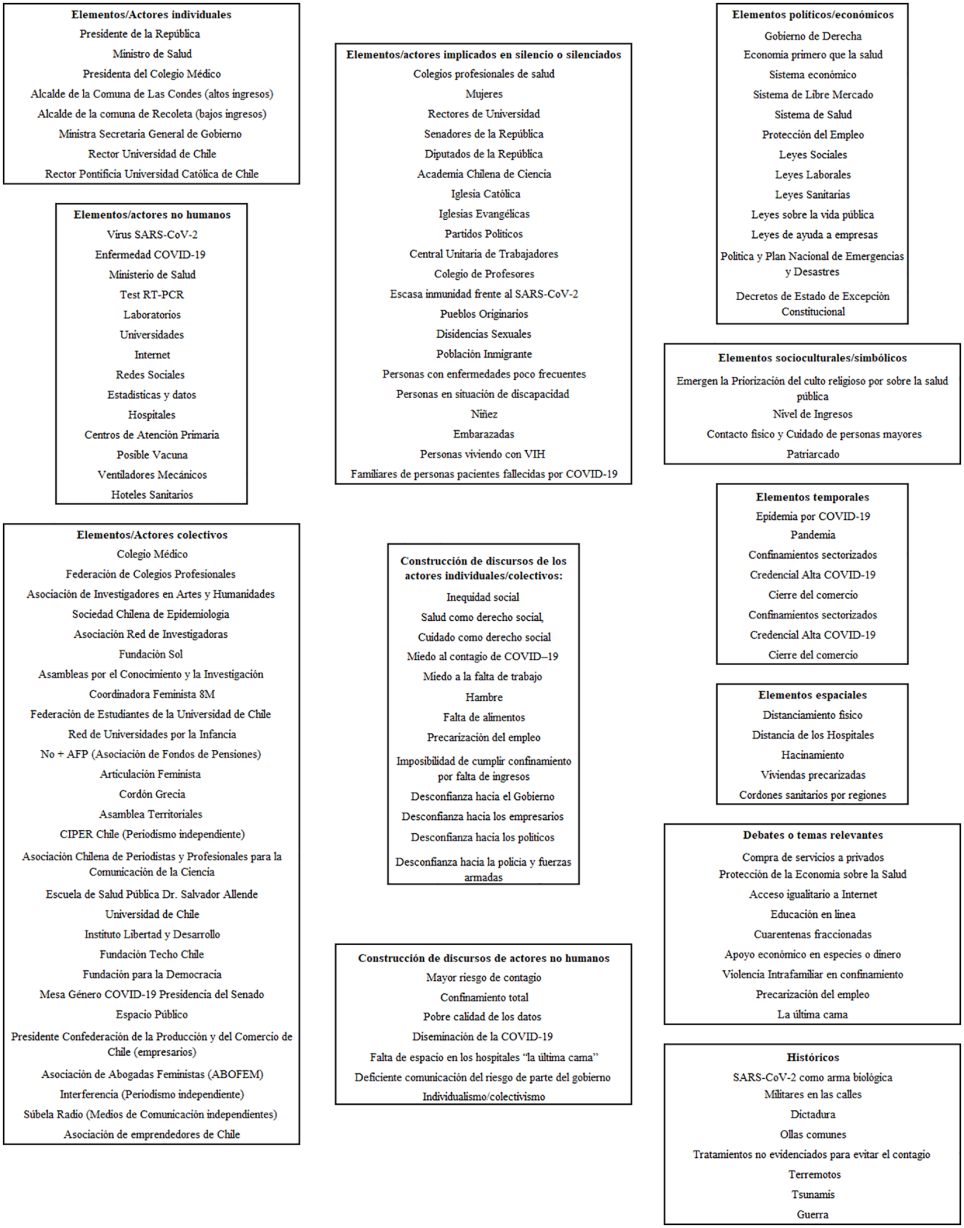
Mapa Situacional de políticas públicas en Chile de promoción de salud en el contexto de la COVID-19.

El Mapa Situacional se confeccionó siguiendo las recomendaciones de Clarke. Se comenzó con un mapa desordenado, el cual incorpora todos los elementos humanos y no humanos (25). En esta investigación, el mapa desordenado se reordenó según las categorías sugeridas por Clarke, las cuales se presentan a continuación (Figura 1):

**Categoría 1 – Elementos/Actores individuales:** Esta categoría incorpora a todos aquellos elementos o actores de tipo individual, cuyo discurso ha estado presente en la discusión política o ciudadana. En esta categoría se incluyen el Presidente de la República, el Ministro de Salud, la Presidenta del Colegio Médico, el Alcalde de la Comuna de Las Condes (comuna de altos ingresos económicos), el Alcalde de la comuna de Recoleta (zona de bajos ingresos económicos), la Ministra Secretaria General de Gobierno, el Rector Universidad de Chile, y el Rector Pontificia Universidad Católica de Chile. De los actores clave identificados, solo el Alcalde Recoleta y la Presidenta del Colegio Médico no son oficialistas. Por otro lado, en el caso de los rectores de universidades, se presentan con mayores menciones principalmente porque fueron los únicos convocados por el gobierno para integrar la Mesa Social COVID-19, correspondiendo además a las universidades más importantes del país en término de antigüedad y prestigio, ambas ubicadas en la ciudad capital.
**Categoría 2 – Elementos/actores no humanos:** En esta categoría se incluyen equipamientos, insumos, instituciones, infraestructura, mundo virtual, entre otras. En esta categoría están presentes: virus SARS-CoV-2, la enfermedad COVID-19, Ministerio de Salud, Test RT-PCR, Laboratorios, Universidades, Internet, Redes Sociales, Estadísticas y datos, Hospitales, Posible Vacuna, Ventiladores Mecánicos y Hoteles Sanitarios.
**Categoría 3 – Elementos/Actores colectivos:** En esta categoría se incluyen los actores colectivos, organizados que conforman el capital social, como así también aquellos que son parte de la institucionalidad gubernamental. Los elementos y actores incluidos en esta categoría fueron Colegio Médico, Federación de Colegios Profesionales, Asociación de Investigadores en Artes y Humanidades, Sociedad Chilena de Epidemiología, Asociación Red de Investigadoras, Fundación Sol, Asamblea por el Conocimiento y la Investigación, Red de Universidades por la Infancia, No + AFP (Asociación de Fondos de Pensiones), Articulación Feminista, Asambleas Territoriales, CIPER Chile (Periodismo independiente), Asociación Chilena de Periodistas y Profesionales para la Comunicación de la Ciencia, Escuela de Salud Pública Dr. Salvador Allende, Universidad de Chile, Instituto Libertad y Desarrollo, Mesa Género COVID-19 Presidencia del Senado, Espacio Público, Presidente Confederación de la Producción y del Comercio de Chile (empresarios), Asociación de Abogadas Feministas (ABOFEM), Interferencia (Periodismo independiente) y Súbela Radio (Medios de Comunicación independientes).
**Categoría 4 – Elementos/actores implicados en silencio o silenciados:** Esta categoría da cuenta de los actores o elementos que no han tenido voz en la discusión de la política pública, aunque estén relacionados directa o indirectamente con ella. Se incluyen aquellos que voluntariamente no han expresado opinión, como así también aquellos que han sido silenciados. En esta categoría se incluyen los Colegios profesionales de salud, a excepción del Colegio Médico, Mujeres, Rectores de Universidades que no son partícipes de la mesa de social COVID-19 y pertenecientes a provincias distintas a Santiago de Chile, Senadores de la República, Diputados de la República, Academia Chilena de Ciencia, Iglesia Católica, Iglesias Evangélicas, Partidos Políticos, Central Unitaria de Trabajadores, Colegio de Profesores, Escasa inmunidad frente al SARS-CoV-2, Pueblos Originarios, Disidencias Sexuales, Población Inmigrante, Personas con enfermedades poco frecuentes, Personas en situación de discapacidad, Niños y Niñas, Embarazadas, Personas viviendo con virus de inmunodeficiencia humana (VIH), Familiares de personas fallecidas por COVID-19.
**Categoría 5 – Construcción de discursos de los actores individuales/colectivos:** Esta categoría ayuda a descifrar los argumentos en relación con la COVID-19. Se incluyen conceptos, símbolos, ideas, discursos, debates, entre otros, que pueden apoyar la comprensión del problema.
En esta categoría emergen los siguientes elementos: Inequidad social, Salud como derecho social, Cuidado como derecho social, Miedo al contagio de COVID-19, Miedo a la falta de trabajo, Hambre, Falta de alimentos, Precarización del empleo, Imposibilidad de cumplir confinamiento por falta de ingresos, Desconfianza hacia el Gobierno, Desconfianza hacia los empresarios, Desconfianza hacia los políticos, Desconfianza hacia la policía y fuerzas armadas.
**Categoría 6 – Construcción de discursos de actores no humanos:** En esta categoría se intenta rescatar los argumentos institucionales que pueden ayudar a comprender las políticas públicas de COVID-19. Se incluyeron los siguientes códigos: Mayor riesgo de contagio, Confinamiento total, Pobre calidad de los datos, Diseminación de la COVID-19, Falta de espacio en los hospitales “la última cama”, Deficiente comunicación del riesgo de parte del gobierno, Individualismo/colectivismo.
**Categoría 7 – Elementos políticos/económicos:** En esta categoría se incluyen los elementos de orden político y económico relacionados con las políticas públicas frente a la COVID-19 En este enunciado emergen los siguientes códigos: Gobierno de Derecha, Economía primero que la salud, Sistema económico, Sistema de Libre Mercado, Sistema de Salud, Protección del Empleo, Leyes Sociales, Leyes Laborales, Leyes Sanitarias, Leyes sobre la vida pública, Leyes de ayuda a empresas, Política y Plan Nacional de Emergencias y Desastres, Decretos de Estado de Excepción Constitucional.
**Categoría 8 – Elementos socioculturales/simbólicos:** En esta categoría se incluyen aquellos elementos propios de la cultura del país, incluyendo los símbolos. Emergen la Priorización del culto religioso sobre la salud pública, Nivel de Ingresos, Contacto físico **y** Cuidado de personas mayores y el Patriarcado.
**Categoría 9 – Elementos temporales:** Los elementos temporales son los aspectos que modifican la libre planificación y están representados por los siguientes códigos: Epidemia por COVID-19, Pandemia, Confinamientos sectorizados, Credencial Alta COVID-19, Cierre del comercio.
**Categoría 10 – Elementos espaciales:** En esta categoría aparecen los elementos que reconfiguran los espacios físicos, la conectividad y/o los espacios de convivencia durante la pandemia describiendo: el Distanciamiento físico, Distancia de los Hospitales, Hacinamiento, Viviendas precarizadas, Cordones sanitarios por regiones.
**Categoría 11 – Debates o temas relevantes:** En esta categoría se presentan aquellos temas que emergen como relevantes para la comprensión del fenómeno y que suscitan discusiones desde las distintas voces del país. Entre ellos se cuentan: Compra de servicios a privados, Protección de la Economía sobre la Salud, Acceso igualitario a Internet, Educación en línea, Cuarentenas fraccionadas, Apoyo económico en especies o dinero, Violencia Intrafamiliar en confinamiento, Precarización del empleo, La última cama.
**Categoría 12 – Discursos de tipo histórico:** En esta categoría se incluyen todos aquellos códigos vinculados con la historia y representaciones sociales en Chile. Los códigos que emergen son: SARS-CoV-2 como arma biológica, Militares en las calles, Dictadura, Ollas comunes (comedores populares), Tratamientos no evidenciados para evitar el contagio (beber alcohol, tomar sol, tomar agua con sal, fumar, entre otros), Terremotos, Tsunamis, Guerra.

## Discusión

Las políticas públicas analizadas se desagregaron en medidas como el cierre de teatros, cines, restaurantes, centros nocturnos y centros deportivos (27), cuarentenas parciales o con fraccionamiento geográfico (28), penas en dinero o cárcel a quienes no cumplan con el confinamiento (29), recomendación de uso de mascarillas en lugares que presentan hacinamiento (30), suspensión transitoria de clases, con el propósito del retorno en breve plazo (31). También se intencionó la compra de ventiladores mecánicos, aumento de camas de unidades de cuidados críticos y capacitación del personal, decisiones en las cuales primó la mirada hospitalocéntrica (32). El Gobierno adelantó la vacunación contra la influenza en población de riesgo, con el fin de evitar la coexistencia de ambos virus (33). El 18 de marzo, con 238 personas infectadas, el gobierno de Chile indicó el cierre de centros comerciales, permitiendo el funcionamiento de supermercados, farmacias, estaciones de gasolina y bancos (34). Dentro de las medidas económicas adoptadas por la presidencia se utilizó el 2% constitucional, equivalente al 4,7% del Producto Interno Bruto (PIB) para enfrentar las consecuencias económicas de la pandemia, lo que se tradujo en un bono a las familias beneficiarias del Subsidio Único Familiar, en las medidas de “protección del empleo”, según las cuales, las empresas pueden suspender el sueldo de los trabajadores durante 3 meses, suspensión por 3 meses de impuestos en favor de las empresas e ingreso familiar de Emergencia destinado al 90% más vulnerable (35). El día 15 de mayo, habiendo transcurrido más de 70 días desde el primer paciente confirmado por COVID-19, se decretó cuarentena total en Santiago (36). Pese a la Declaración de Estado, el estado de excepción constitucional de catástrofe por calamidad pública en todo el territorio nacional (Decreto Supremo 104 de 18 de marzo de 2020), no fue posible constatar documentos o protocolos sobre la activación de la Política y Plan Nacional de Emergencias y Desastres, como tampoco indicaciones o protocolos referidos al Nivel Primario de Atención de Salud y articulación respectiva con las Secretarías Regionales del Ministerio de Salud, instancias institucionales que podrían haber colaborado en fortalecer una respuesta comunitaria, intersectorial, transdisciplinaria y con enfoque preventivo y de mitigación de la emergencia sanitaria y social.

Al analizar las políticas implementadas a la luz de los elementos en el mapa situacional es posible observar el discurso de los diferentes actores. Los actores individuales oficialistas representados por el Presidente de la República, los Ministros de Salud y la Secretaría General de Gobierno, el Alcalde de la zona de mayor ingreso per cápita, con alta presencia en los medios de comunicación masivos, han expresado un discurso alineado con el gobierno, primando un discurso con una comunicación del riesgo inadecuada, con lenguaje bélico, afirmando que “el país estaba en guerra”, pero “mejor preparado que Italia o España” y con ofrecimiento de ayuda a China, en palabras del Presidente (37). Por otro lado, el Ministro de Salud, quien renunció el pasado 13 de junio a su cargo, durante su permanencia mantuvo un estilo confrontacional contra la academia, científicos, la Presidenta del Colegio Médico y la sociedad civil en general, quienes han insistido en la necesidad de transparentar las cifras reales de casos y defunciones y de instalar medidas preventivas y de testeo (38). Los actores colectivos han mantenido una posición contraria a las políticas del gobierno, por la falta de transparencia, establecimiento tardío de confinamiento total y priorización de medidas de tipo económico; proteccionistas de empresas, sobre la vida de las personas, instaurando la idea de “nueva normalidad”, para estimular el regreso a actividades laborales y sociales productivas. El gobierno de Chile ha presionado continuamente con el retorno a clases presenciales de estudiantes de educación primaria y secundaria. La Red de Universidades por la Infancia, conformada por universidades chilenas que tienen como compromiso la protección de los derechos de la niñez, ha representado una voz disidente importante en relación con la premisa de que no existen las condiciones para un retorno seguro a clases (39). Los medios informativos independientes han cumplido un rol fundamental en la transparencia de información y revelación de cifras reales, quienes por medio de la publicación de investigaciones acerca del exceso de muertes, obligaron al Ministerio de Salud a cambiar la forma de contabilización de casos sospechosos, casos probables y defunciones (40). Actualmente el Presidente de la Republica y el ex Ministro de Salud de Chile enfrentan una querella judicial por cuasidelito de homicidio y denegación de auxilio por las muertes en la pandemia. Se presume que, durante los tres primeros meses de la epidemia, se reportó casi un 70% menos de muertes. Las denuncias de manipulación de las cifras y la observación de la cotidianeidad, con familiares y vecinos enfermos y muertos, se tradujo en incertidumbre y miedo en la población.

El Gobierno de Chile es un gobierno favorecedor de la economía de libre mercado. Según esto, resulta coherente la intención de hacer prevalecer la economía, privilegiando a los empresarios y silenciando a los actores disidentes, como es el caso de los pueblos originarios, minorías sexuales, personas de bajo ingresos, obligados a salir diariamente del confinamiento para generar recursos, debido a la precarización del empleo (41).

Chile posee una cultura en que la familia es el pilar de la sociedad, con respeto hacia las personas mayores quienes cumplen en muchos casos labores de cuidado de niños y personas en situación de discapacidad. También está presente el hábito del contacto físico con abrazos y saludo, aumentando de esta forma el riesgo de contagio de COVID-19. En ese mismo sentido, es interesante mencionar la falta de credibilidad del Gobierno y la deficiente comunicación del riesgo, con información confusa e imprecisa, por ejemplo afirmaciones como el “virus se puede convertir en una buena persona”, complejizando aún más el cumplimiento de las medidas preventivas, como son el confinamiento y el distanciamiento físico (42,43).

Otro aspecto interesante de mencionar es la solidaridad, ligada históricamente a los terremotos, tsunamis y a la dictadura. Esto ha permitido una rápida organización ciudadana de preparación de alimentos en los comedores populares u “ollas comunes”, que reciben donaciones para preparar diariamente almuerzos (44).

## Conclusiones

El análisis situacional permite el visibilizar a todos los actores, incluyendo a las minorías silenciadas, relevando sus espacios reivindicativos. Siguiendo con esta misma idea, se observa una fuerte presencia masculina en las distintas categorías y de centralismo geográfico en la toma de decisiones, excluyendo la voz de regiones, mujeres, disidencias, niños, niñas, personas en situación de discapacidad y con enfermedades poco frecuentes. Se observa una predominancia de medidas económicas sobre las sanitarias y una deficiente comunicación del riesgo, además de brechas en la aplicación del Plan Nacional de Emergencias y Desastres.

Como recomendaciones a un mejor manejo de la política pública en pandemia, el equipo investigador considera que es fundamental la transparencia y calidad de los datos para una adecuada respuesta institucional, como así también la creación de espacios de participación ciudadana con representación de todos los actores sociales, énfasis en la prevención y promoción, comunicación adecuada del riesgo, fortalecimiento del tejido social, de la resiliencia comunitaria y el socio-cuidado. Conjuntamente con las actividades antes mencionadas, es primordial terminar con la desigualdad social y la precarización laboral, lograr que la salud sea un derecho y aprobar una nueva constitución que garantice la justicia y la equidad.
